# COVID-19: Impact on the HIV and Tuberculosis Response, Service Delivery, and Research in South Africa

**DOI:** 10.1007/s11904-021-00588-5

**Published:** 2022-01-22

**Authors:** Quarraisha Abdool Karim, Cheryl Baxter

**Affiliations:** 1 Centre for the AIDS Programme of Research in South Africa (CAPRISA), 2nd Floor Doris Duke Medical Research Institute, Nelson R Mandela School of Medicine, University of KwaZulu-Natal, Private Bag X7, CongellaDurban, 4013 South Africa; 2 Department of Epidemiology, Columbia University, New York, NY USA

**Keywords:** SARS-CoV-2, COVID-19, HIV, Tuberculosis, Services, Disruptions

## Abstract

**Purpose of Review:**

To describe how mitigation measures against COVID-19 have impacted HIV and TB research in South Africa.

**Recent Findings:**

South Africa has the highest number of COVID-19 (34%) cases in Africa, accounting for 43% of all reported COVID-19-related deaths on the continent. The country accounts for 20% of all people living with HIV and ranked third in the world for new TB infections in 2019.

**Summary:**

While South Africa’s investments in its HIV and TB responses enabled it to pivot rapidly to respond to the emerging COVID-19 epidemic, it negatively impacted the HIV and TB response through temporary suspension of research, diversion of key resources for HIV and TB control, and patient access to health care facilities; the full extent of this has yet to emerge. Success in integrating responses to the colliding epidemics could potentially enhance survival outcomes and ensure gains made to date in HIV and TB are not reversed and we stay on track toward achieving the UN 2030 Sustainable Development Goals.

## Introduction

### The Burden of COVID-19, HIV, and Tuberculosis in South Africa

**Fig. 1:**
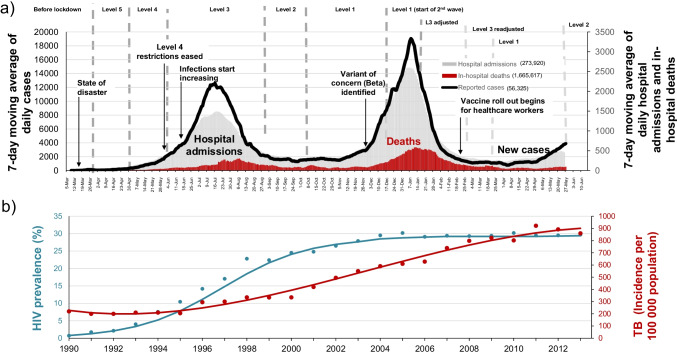
(a) COVID-19 in South Africa: 7-day moving average of new cases, 5 March 2020 to 31 May 2021. Source of data on cases – Department of Health; (b) HIV and TB epidemics in South Africa. Source of data—annual point estimates from the South African Department of Health; http://www.tbfacts.org/tb-statistics-south-africa/ and . The lines are based on fitted mathematical models developed by E Gouws (HIV) and A Grobler (TB)

South Africa has the highest burden of COVID-19 in Africa, accounting for 34% of all cases and 43% of all COVID-19-related deaths on the continent [1]. The first case of COVID-19 in South Africa was reported on 5 March 2020 in a traveler who had recently returned from Italy. The average daily COVID-19 cases peaked at 12,500 in mid-July 2020 in the first wave and at 19,000 in January 2021 in the second wave and are currently on the brink of a third wave. Significantly, the demographic characteristics of hospitalized patients have varied with each wave with over 60-year-old persons with co-morbidities dominating in the first wave shifting to 40–60 year-olds with co-morbidities in the second wave and currently a much younger population bearing the brunt of infections. Interestingly, there have also been geo-spatial differences in terms of burden of infection with large urban cities more severely affected in the first wave; the coastal provinces of Western and Eastern Cape and KwaZulu-Natal dominating in the second wave and currently the inland provinces including the Northern Cape, Free State, and Gauteng are leading the third wave. By May 2021, South Africa had reported more than 1.6 million COVID-19 cases and over 57,000 COVID-19-related deaths [2•] (Fig. 1a). Based on recorded excess deaths in the country over the past year, the actual number of COVID-19 deaths is probably substantially higher [3]. The COVID-19 epidemic in South Africa has unfolded against a backdrop of the substantial and long-standing HIV and TB epidemics.

Despite being home to 0.7% of the world’s population, South Africa accounts for a disproportionate 20% of people living with HIV (PLHIV) globally and a third of all new HIV infections in southern Africa in 2019 [4, 5]. In 2019, there were an estimated 7.5 million South Africans living with HIV, 200,000 new HIV infections, and 72,000 deaths from AIDS-related illnesses [6]. There is a gradient of HIV infection across South Africa, with the east coast provinces having the highest rates of HIV and those on the west coast the lowest. As in most of eastern and southern Africa, young women (15–24 years) acquire HIV about 5–7 years before their male peers, and 1 in 4 new infections are in young women 15–24 years. South Africa has the world’s largest antiretroviral treatment (ART) program and has been making good progress toward the UNAIDS 90–90-90 targets, particularly with HIV testing and viral suppression. There are gender differences in treatment access with women being eight times more likely to be on ART compared to men. In 2019, 92% of people living with HIV in South Africa were aware of their status, of whom 70% were on ART and 64% were virally suppressed [6]. The increased access to ART has resulted in a 61% reduction in AIDS-related deaths between 2010 and 2019 [6]. Men are, however, more likely than women to die from AIDS-related causes. Notwithstanding, there has been a steady increase in life expectancy since 2005 when ART was introduced in public sector health facilities.

The HIV and TB epidemics are closely intertwined (Fig. 1b). Tuberculosis (TB) is the most common opportunistic infection associated with advancing HIV disease in Africa and despite increasing ART access, South Africa ranks among the top three countries in the world in terms of new TB cases and HIV-TB co-infection [7]. In 2019, South Africa had an estimated 360,000 cases of active TB (incidence: 615/100,000 population), of which 14,000 cases were multi-drug-resistant (MDR)-TB. The overall TB treatment success rate in 2019 was 71% for new and relapsed TB and 60% for MDR-TB [7]. TB and HIV remain leading causes of years of life lost in South Africa (Table 1). The demographic characteristics of TB patients mirror that of AIDS patients. In 2019, an estimated 58,000 people died of TB—62% were among TB-HIV co-infected individuals [7]. Of note is that over the past decade, there has been a 50% reduction in TB death rate in South Africa from 224 per 100,000 population in 2010 to 110 per 100,000 population in 2018 [8]. The reductions in new HIV treatment initiations are likely to impact the gains in TB caseload reductions. Integration of HIV and TB services is still at a nascent stage.

**Table 1:** Leading cause of years of life lost in South Africa, 1997, 2007, and 2017

Cause of death	2017		2007		1997	
	Rank	%	Rank	%	Rank	%
HIV/AIDS	1	12.7	3	11.1	4	5.7
Tuberculosis	2	9.6	1	17.5	1	9.9
Lower respiratory infections	3	6.7	2	13.0	2	6.9
Cerebrovascular disease	4	5.1	5	3.8	5	5.5
Diabetes mellitus	5	4.0	-	-	-	-
Hypertensive heart disease	6	4.0	7	2.7	9	3.2
Ischemic heart disease	7	3.8	6	2.7	7	4.7
Endocrine nutritional blood immune	8	3.8	-	-	-	-
Interpersonal violence	9	3.5	10	2.3	8	4.2
Mechanical force	10	3.2	-	-	10	2.6
Diarrheal diseases	-	-	4	10.1	3	6.3
Road injuries	-	-	8	2.5	6	5.4
Meningitis/encephalitis	-	-	9	2.4	-	-

Source: data adapted from Neethling et al. 2020 [9]

The limited knowledge of SARS-CoV-2 transmission dynamics and pathogenesis in March 2020 as well as the high COVID-19 morbidity and mortality rates and overwhelming of health care facilities observed in other countries experiencing epidemics influenced the rapid and stringent responses to COVID-19 in South Africa to slow down the spread of SARS-CoV-2 and gain some time to prepare health facilities to deal with the anticipated care burden.

Here, we describe how infrastructure and human resource investments in responding to the HIV and TB epidemics in South Africa enhanced the country’s response to COVID-19 and how the mitigation measures against COVID-19 have impacted HIV and TB research, resource allocation for HIV and TB control, and health service usage. We also reflect on population differences in vulnerabilities to SARS-CoV-2 compared to HIV and TB in South Africa.

#### Responding to the COVID-19 Epidemic in South Africa

Over the past two decades, South Africa with support from, among others, the Global Fund to fight AIDS, Tuberculosis and Malaria (GFATM) and the United States of America Presidential Emergency Program for AIDS Relief (PEPFAR) has made substantial investments in human resources and equipment for the diagnosis, treatment, and management of HIV and TB. Thus, the country had well-developed infrastructure and experienced health care workers for the management of infectious diseases particularly for HIV and TB that enabled the country to rapidly pivot to respond to the newly emerging COVID-19 epidemic. In addition, through investments from the US National Institutes of Health (NIH) and European and Developing Countries Clinical Trials Partnership (EDCTP), a strong clinical research and regulatory oversight infrastructure was in place to undertake COVID-19 vaccine and treatment trials.

In January 2020, two months prior to the first reported cases of SARS-CoV-2 in South Africa, existing HIV viral load testing platforms were rapidly repurposed for the diagnosis of SARS-CoV-2. Although the surveillance and criteria for and diagnostic testing capacity was initially centralized at the National Institute for Communicable Diseases (NICD), which is part of the National Health Laboratory Services that has previously undertaken epidemic outbreak investigations, by April 2020, the diagnostic testing was decentralized to private and public pathology laboratories throughout the country. Both the TB and HIV diagnostic platforms have been central to COVID-19 diagnostic testing. Polymerase chain reaction (PCR) platforms used for HIV viral load testing, as well as the GeneXpert point-of-care testing platform, widely used in South Africa to diagnose TB [10], became the essential diagnostic platforms for SARS-CoV-2 detection. Protocols to perform whole genome sequencing [11, 12•] and phylogenetic analysis to enhance epidemiological outbreak investigations were also established by January 2020 building on HIV molecular surveillance systems established in 2016 to advance HIV transmission dynamics. In February 2020, several public sector facilities in all nine provinces were identified as COVID-19 hospitals with infection control measures in place; appropriate patient screening and triaging and dedicated wards with trained staff to isolate and manage for persons under investigation (PUI) for COVID-19 in the public sector. SARS-CoV-2 was included in the list of notifiable conditions and the criteria for SARS-CoV-2 diagnostic testing until April 2020 was recent travel outside South Africa or contact with a PUI for SARS-CoV-2 [13].

On March 5, when the first case of COVID-19 was reported, the government ramped up its response to curtail the community spread of SARS-CoV-2 infections. A national state of disaster was declared on March 15 (10 days after the first case was reported) that restricted international travel, closed schools, limited public gatherings, and introduced a mandatory daily curfew between 9 pm and 5 am [13]. On March 27, when there were 402 reported SARS-CoV-2 cases, a national mandatory lockdown was instituted, restricting the movement of people except for a few categories of essential staff, including health care workers.

The national lockdown was a mitigation strategy to slow the spread of the virus and was used to prepare for the surge of cases by strengthening health care facilities particularly in the public health sector, strengthen the diagnostic and clinical care capacity, build field hospitals, secure personal protection equipment, and secure medical oxygen supplies to better cope with the anticipated increase in the number of hospital admissions. The government also established a Ministerial Advisory Committee for COVID-19 (Covid-19 MAC) that provided high-level strategic advice to the Minister of Health and evidence-based guidance to the National Department of Health. The inaugural COVID-19 MAC that was dissolved in September 2020 included 50 of South Africa’s leading clinicians, virologists, epidemiologists, mathematical modelers, and public health practitioners, the majority of whom are also South Africa’s foremost TB and HIV scientists.

During the mandatory lockdown in April 2020, the focus of South Africa’s response shifted to scaling up community-based COVID-19 screening and testing. The community-based screening approach drew heavily on the country’s experiences in managing the TB and HIV epidemics. The country’s extensive network of several thousand community workers was deployed to undertake COVID-19 symptom screening, with referral for testing, in vulnerable high-density communities [14]. However, as restrictions started easing and clinical cases increased, there were insufficient test kits (due to global shortage in supply) for community-based screening creating testing backlogs that delayed hospital patient results leading to the curtailment and adjustment of the community program to screening and quarantine without testing [13].

#### Impact of COVID-19 on HIV Services

Mitigation measures against COVID-19, such as travel bans and national lockdowns, have negatively impacted HIV care and treatment globally. According to the World Health Organization (WHO), 73 countries worldwide experienced disruptions to their antiretroviral therapy (ART) programs between April and June 2020, potentially impacting 70% of people receiving ART [15]. Travel restrictions and flight cancelations affected the supply and transportation of medicines [16] while national lockdowns restricted people’s movement, leading to substantial declines in patient attendance at health care facilities and declines in HIV testing and monitoring [17]. The WHO estimates that a six-month disruption of ART could lead to more than 500,000 additional deaths from AIDS-related illness in 2021 and a reversal of gains made in the prevention of mother-to-child transmission [18].

In South Africa, during the mandatory national lockdown (level 5—27 March – 30 April 2020), where all non-essential services were closed, access to medical care for non-COVID-19 conditions was limited. A national survey (*n* = 19,330) assessing communities’ knowledge, beliefs, practices, and attitudes in response to the COVID-19 outbreak in South Africa found that 13.2% of people were unable to access medication for their chronic disease during the lockdown [19]. Furthermore, hospital admissions for HIV declined because of hospitals reducing non-urgent admissions in preparation for a surge of COVID-19 cases and some facilities closed to reduce exposure to COVID-19 patients. Fear of being exposed to SARS-CoV-2 from COVID-19 patients attending these facilities made some people reluctant to visit a clinic or hospital during the lockdown [20].

Data from the National Health Laboratory Services shows that, compared to pre-lockdown periods, the average weekly HIV-1 viral load testing in South Africa declined by 22% and CD4 + cell count testing declined by 33% during the lockdown [21••]. An analysis of data from 65 primary care clinics in the province of KwaZulu-Natal in South Africa, where 1.7 million people are living with HIV, revealed that HIV testing decreased by 47.6% and ART initiations declined by 46.2% in April 2020 [22••]. Delays in HIV testing impeded initiation of, or access to ART, which potentially increased the risk of new infections and emergence of drug resistance through treatment interruptions [23]. However, as restrictions eased, HIV testing and ART initiations gradually improved toward pre-lockdown levels suggesting that the disruptions to HIV testing and ART initiations were temporary. Fortunately, ART provision was generally maintained during the lockdown through multi-month drug dispensing and innovative treatment delivery strategies in place to manage the growing number of patients on ARVs and had been expanded for other chronic conditions. In the 65 clinics in KwaZulu-Natal, the median number of ART collection visits before lockdown was 18,519 and after lockdown was 17,863. Telemedicine platforms were another strategy that was successfully utilized in several settings to continue provision of healthcare to PLWH and reduce exposure to COVID-19 [24–26].

#### Impact of COVID-19 on TB Services

Disruptions to TB programs caused by the COVID-19 pandemic also threaten to reverse recent progress in reducing the global TB burden. Modeling data indicate that a 25–50% decline in the number of people diagnosed and treated for TB over a period of 3 months could lead to an additional 400,000 TB-related deaths in 2020. The GFATM survey that included 106 countries found that 78% of countries experienced disruptions to TB services as a result of COVID-19 in 2020, with 17% of countries experiencing high or very high disruptions [27]. Some of the factors contributing to the disruptions of TB programs included people avoiding seeking care, reductions in the number of health facilities offering TB diagnostic and treatment services, reallocation of human and financial resources, repurposing of diagnostic platforms to the COVID-19 response, a disruption in the procurement, supply and transportation of medicines and laboratory consumables, and restrictions in movement and loss of wages making it harder for people to travel to health facilities [28]. A decrease in the number of TB cases diagnosed and sub-optimal adherence and support during TB treatment could result in poorer treatment outcomes and consequently increase transmission that would increase the TB burden and TB-related mortality [29].

In South Africa, monthly TB notifications decreased by more than 50% between March and June 2020 [21••]. The average number of weekly TB tests declined from 49,109 during the 7 weeks prior to lockdown to an average of 24,620 during lockdown. During the same time periods, the weekly average of microbiologically confirmed TB cases declined by 33% from a weekly average of 3,707 to 2,465 [21••]. Data from the national testing laboratory between 3 February 2020 and 3 May 2020 show that national Xpert MTB/RIF Ultra tests declined by 48% and Xpert positive tests declined by 33% during the lockdown period [30] but rapidly returned to previous levels soon after restrictions were lifted. Therefore, the short-term infection-control measures implemented to reduce COVID-19 transmission may have had limited impact on TB incidence rates.

Actions to mitigate the impact of disruptions to TB services have included the reduction in the frequency of outpatient visits for treatment monitoring or collection of drugs, allowing multi-month drug dispensing, allowing a family member or friend to collect anti-TB drugs for the TB patients, and expanded use of telemedicine to provide advice and support [28].

#### Interaction between COVID-19 and HIV

Early in the COVID-19 epidemic, there were concerns that immunocompromised individuals would be at higher risk of severe COVID-19. With about 7.5 million PLHIV in the country, there was a concern that immunocompromised individuals would be disproportionately affected by COVID-19. Thus far, the data on the impact of HIV on COVID-19 outcomes have been contradictory. Although some studies indicate that HIV-infected individuals with COVID-19 have similar clinical and radiological presentation and outcomes as the general population, others demonstrate an increased risk of death associated with COVID-19 in HIV-infected individuals. Most of the studies have been small cohort studies that limit generalizability. A systematic review of 25 studies from 8 countries including 252 individuals found that PLHIV and COVID-19 had similar features of disease risk and progression as HIV-uninfected individuals [31]. This analysis demonstrated that, among the HIV-infected patients who died, 91% were 50 years old or older, 86% were male, and 64% had co-morbidities. Therefore, older age and co-morbidities are important factors contributing to excess morbidity and mortality in individuals co-infected with COVID-19 and HIV [31].

Two studies have shown an increased risk of COVID-19 mortality among PLHIV. A population cohort study (*n* = 22,308) from the Western Cape in South Africa, which assessed the impact of HIV and risk of COVID-19 death, revealed that HIV increased the risk of COVID-19 mortality by about twofold. The standard mortality ratio for COVID-19 death associated with HIV was 2.39 (95% CI:1.96; 2.86); population attributable fraction 8.5% (95%CI: 6.1; 11.1) [32••]. This figure is comparatively modest against the expected COVID-19 deaths in PLHIV and the actual COVID-19 deaths associated with other co-morbidities such as diabetes and hypertension [33]. A retrospective cohort study from the UK that included 27,480 PLHIV showed that compared to HIV-uninfected individuals, PLHIV had a threefold higher risk of COVID-19 death (HR: 2.90 (95% CI 1.96–4.30; *p* < 0·0001) with some evidence that the association was larger among people of Black ethnicity [34•].

Individually and collectively, these studies do not provide sufficient data to understand the relationship between COVID-19 and HIV at a clinical or epidemiological level. It is therefore important to establish, using larger data sets, the clinical interaction, if any, between COVID-19 and HIV infection.

Of greater concern is the potential impact that immunocompromised individuals could have on the evolution of SARS-CoV-2. Persistent long-term active infection in immunocompromised individuals has been shown to be an important source of new variants [35•, 36••]. This highlights the need to strengthen HIV programs along the treatment continuum, ensuring PLHIV are diagnosed, put on treatment, and maintain viral control, as well as ongoing genomic surveillance to rapidly detect new SARS-CoV-2 variants of concern. With the growing number of new variants being identified across the globe more attention is turning to strengthening molecular surveillance and use of phylogenetic mapping of circulating viruses.

#### Impact on HIV and TB Research

South Africa has well-developed research infrastructure to conduct clinical trials and has contributed to several important studies that have impacted the prevention and treatment of HIV and TB globally. A search in Clinicaltrials.gov revealed that there are currently 611 ongoing HIV or TB clinical trials in South Africa and the Pan African Clinical Trials Registry (PACTR) has 73 ongoing clinical trials on HIV and TB that are being led by South African principal investigators. When the national state of disaster was declared in March 2020, all clinical trials were halted by the research ethics committees in the country. Although research ethics committees fast-tracked all COVID-19 studies, important prevention and treatment studies were placed on hold. Meetings between scientists and regulatory oversight bodies led to a reversal of these restrictions provided that research organizations had strict COVID-19 risk mitigation protocols in place for staff and participants, including making changes to the physical lay-out of facility. Some of the adjustments made at research facilities included ensuring appropriate physical barriers were in place between staff and participants with adequate social distancing, physical separation of COVID-19 and HIV research spaces, availability of information on COVID-19 in the clinic waiting areas, establishment of a designated isolation room for participants presenting with COVID-19 symptoms, mandatory daily screening of COVID-19 symptoms of staff, and ensuring adequate supplies of PPE were available at the research sites and the compulsory wearing of masks by all participants and research study staff. Where possible, interactions with participants were done telephonically, and all community engagements were used as opportunities for education and sharing of information on COVID-19. All participants were screened telephonically prior to arriving at the research site, and all research vehicles transporting participants were sanitized after each trip. Mask wearing was compulsory for everyone in the research vehicles.

Not surprisingly, the South African research infrastructure and capacity is being utilized for COVID-19 research and includes studies to understand transmission dynamics; pathogenesis; evaluate candidate vaccines and therapeutic agents; and undertake molecular surveillance [37–39]. The national genomics surveillance network that conducts ongoing SARS-CoV-2 genomic surveillance across all provinces enabled the early identification of the more transmissible B.1.351 (501Y.V2) variant of concern [40] that emerged in November 2020 and further facilitated the in vitro impact of the variant of concern on COVID-19 vaccine efficacy.

## Conclusions

SARS-CoV-2 continues to challenge us, and with each surge within and between countries, we learn more about how little we actually know. While the vaccines bring much hope, inequity in terms of access particularly in LMICs while morally and ethically unacceptable in the face of our collective vulnerabilities it also fuels the emergence of variants of concern and undermines the protective benefits from high vaccination coverage rates. The growing cohorts of patients with long COVID-19 with a wide range of co-morbidities underscore the importance of the need for greater investments in COVID-19 treatment efforts. COVID-19 has highlighted our interdependence as a global community but its devastating impact on lives and livelihoods lost has been uneven with those already most vulnerable yet again bearing the brunt.

As we build back stronger to live with COVID-19, we cannot afford to have the HIV and TB pandemics left behind to join the litany of other incomplete pandemics or worse not be better prepared for pandemics yet to emerge. COVID-19 creates new opportunities to accelerate the Sustainable Development Goals. Its unmasking of epidemics of non-communicable diseases in Africa provides an opportunity for more community-based preventive interventions that include enhancing responses for HIV, TB, and Malaria and strengthening linkages to care. As more point-of-care diagnostic tests emerge for COVID-19 antigen detection, it provides an opportunity to strengthen community level testing to enhance early detection of infections and increase knowledge, awareness and screening, and testing and linkages to care for HIV and TB. We have seen how the adoption of COVID-19 infection control practices such as cough etiquette, and the wearing of masks have reduced TB transmission. Molecular surveillance has been an important adjunct for detection of SARS-CoV-2 variants of concern and could be extended for HIV and TB drug resistance monitoring. The COVID-19 vaccine roll-out programs have also highlighted the importance of the urgent need for the modernization of health information systems. Digital divides in terms of who has access to data and internet are widening disparities between the haves and have nots. In the words of the WHO DG, COVID-19 has reminded us that no-one is safe unless we are all safe and it is this global solidarity that will give us the resilience to live with COVID-19 and deal with new and emerging threats and work toward better health for all.
